# A Novel Computational Model for Detecting the Severity of Inflammation in Confirmed COVID-19 Patients Using Chest X-ray Images

**DOI:** 10.3390/diagnostics11050855

**Published:** 2021-05-10

**Authors:** Mohammed S. Alqahtani, Mohamed Abbas, Ali Alqahtani, Mohammad Alshahrani, Abdulhadi Alkulib, Magbool Alelyani, Awad Almarhaby, Abdullah Alsabaani

**Affiliations:** 1 Department of Radiological Sciences, College of Applied Medical Sciences, King Khalid University, Abha 61421, Saudi Arabia; maalalyani@kku.edu.sa; 2 BioImaging Unit, Space Research Centre, Department of Physics and Astronomy, University of Leicester, Leicester LE1 7RH, UK; ama78@leicester.ac.uk; 3 Electrical Engineering Department, College of Engineering, King Khalid University, Abha 61421, Saudi Arabia; mabas@kku.edu.sa; 4 Computers and Communications Department, College of Engineering, Delta University for Science and Technology, Gamasa 35712, Egypt; 5 Medical and Clinical Affairs Department, King Faisal Medical City, Abha 62523, Saudi Arabia; a.m.qahtani@kfmcity.med.sa (A.A.); aalkulib@kfmcity.med.sa (A.A.); 6 Department of Clinical Laboratory Sciences, College of Applied Medical Sciences, King Khalid University, Abha 61421, Saudi Arabia; moyahya@kku.edu.sa; 7 Department of Family and Community Medicine, College of Medicine, King Khalid University, Abha 61421, Saudi Arabia; aalsabaani@kku.edu.sa

**Keywords:** auto-detection, SARS-COV-2, chest X-ray images, lung infection, disease severity

## Abstract

Since late 2019, Coronavirus Disease 2019 (COVID-19) has spread all over the world. The disease is highly contagious, and it may lead to acute respiratory distress (ARD). Medical imaging can play an important role in classifying, detecting, and measuring the severity of the virus. This study aims to provide a novel auto-detection tool that can detect abnormal changes in conventional X-ray images for confirmed COVID-19 cases. X-ray images from patients diagnosed with COVID-19 were converted into 19 different colored layers. Each layer represented objects with similar contrast that could be defined as a specific color. The objects with similar contrasts were formed in a single layer. All the objects from all the layers were extracted as a single-color image. Based on the differentiation of colors, the prototype model was able to recognize a wide spectrum of abnormal changes in the image texture. This was true even if there was minimal variation of the contrast values of the detected uncleared abnormalities. The results indicate that the proposed novel method can detect and determine the degree of lung infection from COVID-19 with an accuracy of 91%, compared to the opinions of three experienced radiologists. The method can also efficiently determine the sites of infection and the severity of the disease by classifying the X-rays into five levels of severity. Thus, the proposed COVID-19 autodetection method can identify locations and indicate the degree of severity of the disease by comparing affected tissue with healthy tissue, and it can predict where the disease may spread.

## 1. Introduction

Since its discovery in Hubei province, China, Coronavirus disease 2019 (COVID-19) has become an international emergency [1,2]. To date, quarantine has been the most significant control intervention for respiratory diseases caused by the virus. Although isolating infected individuals has had positive effects on the distribution of the disease, many more preventive measures have yet to be identified [3,4]. There is currently no cure for COVID-19 that mitigates its global impact on public health and improves the overall ability of healthcare systems to provide adequate care. In addition, the disease increases the need for intensive care, including mechanical ventilation. This has led to the need to redistribute clinical resources for the provision of appropriate care [5,6,7].

In addition to the different clinical procedures and treatments currently available, artificial intelligence (AI) technologies and computer-aided detection and smart diagnostic methods provide a new paradigm for medical settings [8,9,10]. Various automated smart tools that use machine learning algorithms have been used to analyze data sets and enhance various decision-making processes [11]. Computer-aided detection tools could help identify outbreaks of COVID-19 and predict the nature of its spread around the globe [12,13,14]. However, unlike other health issues, to detect COVID-19, AI-driven tools are expected to have active cross-population learning/test models that use a multitude of multimodal data. Testing and isolating positive cases are the most important milestone in managing COVID-19. Diagnosis is currently achieved by a rapid, real-time reverse transcription polymerase chain reaction. This method relies on respiratory samples, and the time to produce results can be two days. As an alternative, the disease can be diagnosed by radiography, which produces ground-like opacities in chest scans of people infected with respiratory diseases. Hazy darkened spots in radiographic images from patients with COVID-19 are different from those of negative subjects. Radiographic analyses have been shown to be useful in the detection, quantification, and follow-up of 19 patients with COVID-19 [15].

X-ray detection of COVID-19 could provide more advantages than conventional polymerase chain reaction (PCR) diagnostic techniques. Moreover, chest X-rays produce results quickly and with greater availability than the PCR test kits. This method is more readily available, and it can be used in installations where there is no adequate supply of PCR test kits [16,17]. As such, radiological diagnostic methods are more convenient given that healthcare is making rapid progress towards radiological imaging techniques in the field of medical diagnosis [18].

In addition to the diagnosis of diseases, medical imaging also provides a wealth of information on the anatomy and physiology of respiratory organs. The integration of imaging methods into medical imaging and machine learning increases the use of computerized diagnostics and decision-making tools. In addition, researchers have seen significant reproducibility and reduced costs from using X-rays for diagnostic purposes compared to conventional test methods [19,20,21].

From a lung computed tomography (CT) scan, the AI is designed to quickly detect lesions of possible coronavirus pneumonia, to measure their volume, shape, and density, and to compare changes in multiple lung lesions from the image. All of this becomes a quantitative report to assist physicians with rapid assessment [22]. Chen [23] adds that, in Wuhan, where there were too many cases to be tested and PCR-based diagnostics took too long, CT imaging with AI may serve as a surrogate for physicians when prompt judgment is needed [24].

Another study investigated how Bayesian convolutional neural networks (BCNN)–based drop-weights can estimate uncertainty in a deep learning solution to improve the diagnostic performance of a human–machine team. Using a publicly available COVID-19 chest X-ray dataset, the study showed that uncertainty in the forecast was highly correlated with the accuracy of the prediction [25]. A three-phase approach has been proposed: the first detects the presence of an X-ray of chest pneumonia; the second distinguishes between COVID-19 and pneumonia; and the third locates the symptomatic X-ray areas of the presence of COVID-19 [26]. A method for generating synthetic chest X-ray images has been introduced by developing a model based on an auxiliary classifier generative adversarial network—called CovidGAN—to enhance the performance of CNNs for COVID-19 detection [27].

Specimens were tested using a validated reverse transcription-quantitative polymerase chain reaction test to detect SARS-COV-2 and measure cycle threshold values. The status of the symptoms and the date of onset of symptoms also were recorded for each participant [28]. Coronavirus was detected using a deep learning model, a sub-branch of AI. Efficient features were combined and classified using vector machine support [29]. A quick and effective way is proposed to identify COVID-19 patients with multitasking deep learning methods. X-ray and CT scan images shall be considered for assessing the proposed technique [30]. A hybrid COVID-19 detection model based on an improved marine predator algorithm has been proposed to segment an X-ray image to reveal similarity in small regions with characteristics of COVID-19 [31]. A novel learning architecture called detail-oriented capsule networks has been proposed for the automatic diagnosis of COVID-19 from computed tomography scans. The network combines the strength of capsule networks with several architectural improvements designed to increase the accuracy of classification [32]. If AI-based intelligence were implemented correctly, it would be far less accurate than that of a human. to precisely, quickly, and rapidly; the missiles could be enhanced with accuracy, precision, and speed.

Utilizing a smart auto-detection computational model that can provide superior accuracy for differentiating abnormalities will enable us to easily distinguish between different cases of COVID-19 X-ray images, and it makes it faster to distinguish features among them. Furthermore, it is vital to identify the abnormal region within the radiological image, as such identification informs potential treatment guidelines for the management of disease symptoms and indicators of acute illness. The aim of this work is to propose the use of image segmentation based on the distribution of texture and intensity in chest X-rays for the effective detection of abnormal locations in chest X-rays. Using this method, the abnormal regions can be identified by texture analysis, depending on the intensity and gradient of the region following the segmentation of the image, which will enhance the possibility of detecting lung complications caused by COVID-19 and provide adequate information to guide quantification and follow-up decisions.

## 2. Materials and Methods

Pulmonary physicians increasingly rely on chest X-rays for diagnosing. However, the conventional segmentation of images is not considered a key element for detecting abnormalities in radiographic images [33]. The viability of disease-based diagnostic methods is, unfortunately, specific to diseases with significant differences. The medical sector has recently seen an increase in the applicability of digital imaging for diagnosing disease. Nevertheless, some techniques used to process medical images are still manually adjusted. Digital processing techniques offer additional advantages, including accuracy, accelerated disease diagnosis, and enhanced test efficiency [34]. Automatic image processing techniques through segmentation may, however, compromise the quality of the image, depending on the type of equipment used and the delivered radiation dose. To address this discrepancy, the current study used multi-scale texture analysis and advanced segmentation tools. The recognition and classification of abnormal regions in the X-ray images could thus be achieved without manual segmentation. Essential features may be retained and distinguished from irregular patches in automated processed images, based on different textures, organ shapes, and pathologies of lung tissue biopsies when automated imaging is used.

This study was approved by the Research Ethics Committee at King Khalid University, Kingdom of Saudi Arabia (Ethical approval code: [ECM#2020-243]—[HAPO-06-B-001]; Approval date: 18 May 2020). In this retrospective study, the proposed methodology was built based on a collection of X-ray images for confirmed COVID-19 patients. Six hundred eighty-nine images were collected from different hospitals in Asir province, Kingdom of Saudi Arabia, twenty-five of which were excluded from the sample due to incorrect positioning (i.e., parts of one or both lungs out of the field of view). The analyzed data included 239 female and 425 male cases. The mean age of these subjects was approximately 55 years, and the standard deviation was ±7.8.

### 2.1. The Prototyped Multicolor Thresholding with Segmentation Model

While designing the computational model used in this study, the validated SARS-COV2 chest X-ray images were chosen as the starting point. Then, the model went through two paths. The first path shows the availability resources and flexible usage. We used an extended nineteen-color (multi-color) path that permitted the use of multiple colors, and a diverse array of representations of the medical images was taken from various patients and assigned unique layer names. When a combination of different objects was shown in each of the colored layers, they were defined as having unique characteristics. All the objects of the same color fit in a single layer. There are many more things to convert, but everything from all the layers had been successfully converted into a single monochrome image. Being able to differentiate between different textures by color allowed the prototype to provide for a large variety of conditions. Since, to the extent that the presence of detectable abnormalities is negligible, the above holds true, even if they have not been fully cleared, the finding is still relevant.

**Figure 1:**
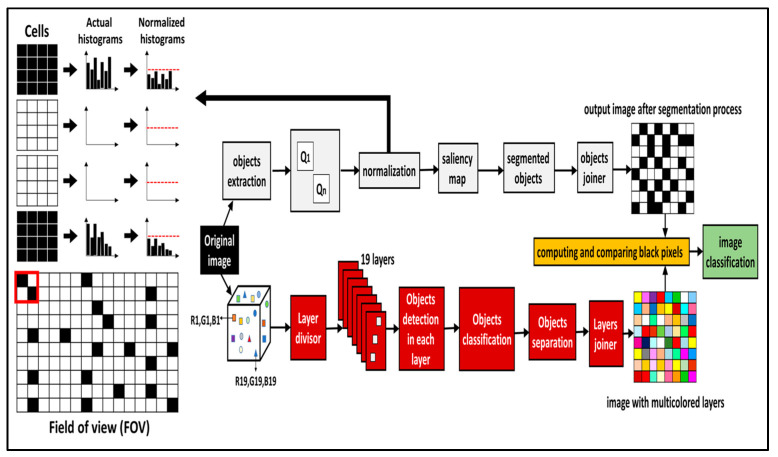
Schematic for the prototyped color-thresholding auto-detection method.

In the second path, according to this more sophisticated method, the patient segmentation technique, whether there is lung expansion in the COVID-19 classification and resale images, is checked. Previously, the image was blurry to enable better detail in color; in this case, we would break up the chest radiographs by image, segmenting the pictures into black and white. Instead, the objects in each X-ray image were expanded and extracted, and then, the dataset was expanded to its optimal size, where it was normalized. To provide a visual map of the highlights of each area, the information included in the outline (the information’s visual properties) was investigated and improved. These aspects were then drawn out in and described as a visual highlight for the area. Thus, the segmented black and white image was recreated by stitching all the images together to form a single, seamless black and white image. A measurement of the abundance of COVID-19 was taken to find the next, by probing possible sources and regions and researching regions and their capacities. It compares the black and white pixels of both images, then produces an intermediate black and white version for comparison, thus simulating the effect of going from one output to the other. X-ray processing in the case of complex object detection can depict multiple portions of the chest, allowing for the systematic and automated processing of X-ray images of object detection (Figure 1). Furthermore, the field of view is the extent of the observable image that is seen at any given moment. Q1 and Qn are the objects in the image, from object No. 1 to object No. n.

The following section explains the core mathematical model for the proposed autodetection method. Using the average method, assume the original image in the grayscale image is as follows:

Grayscale = ((R + G + B)/19)(1)

Equation (1) shows that each color image will be divided into nineteen sub-grayscale images. This division will make it more efficient to deal with the grayscale images. In our proposed methodology, we use 19 colors from wheel 7. This means that the 19 colors will be derived from the seven basic components of a color, which may contain red, blue, yellow, white, black, colorless, and light. Thus, they can be easily monitored and detected using 24-bit color, assuming that the image can be converted to a K × L image histogram with intensity *i*. Then, for each pixel *p*, there is an intensity *i*. Then, *pi* refers to a pixel with its intensity, and the number of pixels is *n*. The image intensity will be the two image dimensions’ matrix and relates them to the surface coordinates, excluding the intensity that fails to be recorded in the image. Then the image intensity (IS) will be defined in terms of the two image dimensions’ matrix (DM) and the intensity that fails to be recorded in the image (IF), as follows:

IS = DM − IF(2)

where the two image dimensions’ matrix equals $\overset{19}{\underset{i=1}{\sum }}M\left(x,y\right)$, and the intensity that fails to be recorded equals $\left(\frac{\partial }{\partial x}\int^{\;}M\left(x,y\right)dy\right)\overset{n}{\underset{m=1}{\sum }}P\left(R+G+B\right)/i{)}_{m}$.

The prototyped method is based on multilevel color-thresholding that segments a greyscale image into several distinct regions by using intensity. This system uses two thresholds to segment the image into certain regions of brightness, which correspond to at least one background and several other objects. We used a layer divisor to convert the resulting image after thresholding it into 19 layers.

Layered images (L) are equal to the sum of all sub-grayscale images, where these partial images could be expressed, as shown in the following expression:

$$\left[\overset{19}{\underset{i=1}{\sum }}P\left(R+G+B\right)/i)\right]$$

where *i* is the number of sub-grayscale images. Now, assume the threshold is *TH* = *m*:*n* and we have 19 layers. The image intensity will be the two image dimensions, and relates them to the surface coordinates, excluding the intensities that fail to be recorded in the image. Then, the probability of the appearance of each layer (*pl*) is calculated as follows:

The general solution of $x=\sqrt{{\mathrm{Pl}}^{//}-\mathrm{Pl}}$

The auxiliary equation is $m^{2}-1=0\Rightarrow m=1$ or m=−1

$${\mathrm{Pl}}^{//}=\overset{m}{\underset{b=0}{\sum }}{\left[\left[\overset{19}{\underset{i=1}{\sum }}\overset{n}{\underset{m=1}{\sum }}P\left(R+G+B\right)/i{)}_{m}\;\right]/s\right]}_{b}{\;}^{//}\;\;\mathrm{Pl}=\overset{m}{\underset{b=0}{\sum }}{\left[\left[\overset{19}{\underset{i=1}{\sum }}\overset{n}{\underset{m=1}{\sum }}P\left(R+G+B\right)/i{)}_{m}\right]/s\right]}_{b}$$

where the value of *s* varies from 1 to 19. The complementary function of the differential equation is

$$y_{c}=k_{1.}e^{\sqrt{{\mathrm{Pl}}^{//}-\mathrm{Pl}}}+k_{2}e^{-\sqrt{{\mathrm{Pl}}^{//}-\mathrm{Pl}}}$$

Let $c_{i}$, (i=1,2) be functions of x:

$$y_{p}=c_{1.}e^{\sqrt{{\mathrm{Pl}}^{//}-\mathrm{Pl}}}+c_{2}\;e^{-\sqrt{{\mathrm{Pl}}^{//}-\mathrm{Pl}}}$$

Differentiate to obtain $y^{/}{}_{p}=c_{1.}e^{\sqrt{{\mathrm{Pl}}^{//}-\mathrm{Pl}}}-c_{2}e^{-\sqrt{{\mathrm{Pl}}^{//}-\mathrm{Pl}}}$ + $c_{1}^{/}e^{\sqrt{{\mathrm{Pl}}^{//}-\mathrm{Pl}}}+c_{2}^{/}e^{-\sqrt{{\mathrm{Pl}}^{//}-\mathrm{Pl}}}$

We have

$$c2=-c1e^{2\sqrt{{\mathrm{Pl}}^{//}-\mathrm{Pl}}}=-\frac{1}{2}x{\sqrt{{\mathrm{Pl}}^{//}-\mathrm{Pl}}}^{2}\;\times \;e^{\sqrt{{\mathrm{Pl}}^{//}-\mathrm{Pl}}}$$

Again, integration by parts yields

$$c_{2}=-\left(1-\sqrt{{\mathrm{Pl}}^{//}-\mathrm{Pl}}+\frac{1}{2}{\left(\sqrt{{\mathrm{Pl}}^{//}-\mathrm{Pl}}\right)}^{2}\right)\;\times \;e^{\sqrt{{\mathrm{Pl}}^{//}-\mathrm{Pl}}}$$

Then, the general solution in terms the pixels of the colored image is, as usual, the sum of the complementary function and the integral, as follows:

$$y\;=\;k_{1.}e^{\sqrt{\overset{m}{\underset{b=0}{\sum }}{\left[\left[\overset{19}{\underset{i=1}{\sum }}\overset{n}{\underset{m=1}{\sum }}P\left(R\;+\;G\;+\;B\right)\;/i{)}_{m}\;\right]/s\right]}_{b}{\;}^{//}-\overset{m}{\underset{b=0}{\sum }}{\left[\left[\overset{19}{\underset{i=1}{\sum }}\overset{n}{\underset{m=1}{\sum }}P\left(R\;+\;G\;+\;B\right)\;/i{)}_{m}\;\right]/s\right]}_{b}\;}}+\;k_{2}e^{-\sqrt{\overset{m}{\underset{b=0}{\sum }}{\left[\left[\overset{19}{\underset{i=1}{\sum }}\overset{n}{\underset{m=1}{\sum }}P\left(R\;+\;G\;+\;B\right)\;/i{)}_{m}\;\right]/s\right]}_{b}{\;}^{//}-\overset{m}{\underset{b=0}{\sum }}{\left[\left[\overset{19}{\underset{i=1}{\sum }}\overset{n}{\underset{m=1}{\sum }}P\left(R\;+\;G\;+\;B\right)\;/i{)}_{m}\;\right]/s\right]}_{b}\;}}-\;{\sqrt{\sum_{b=0}^{m}{\left[\left[\sum_{i=1}^{19}\sum_{m=1}^{n}P\left(R\;+\;G\;+\;B\right)\;/i{)}_{m}\;\right]/s\right]}_{b}{\;}^{//}-\sum_{b=0}^{m}{\left[\left[\sum_{i=1}^{19}\sum_{m=1}^{n}P\left(R\;+\;G\;+\;B\right)\;/i{)}_{m}\;\right]/s\right]}_{b}\;}}^{2}-2$$

The proposed methodology relies on measuring the impact of COVID-19 on the lungs. This effect appears in X-ray images as bright pixels in the lungs’ shadow area. Bright pixels indicate the presence of swelling in the alveoli affected by the virus [35]: the more bright pixels, the more severe the disease. The number of dark pixels in the original image is measured in relation to the total number of pixels in both the original X-ray and the multi-colored X-ray. Then, the difference between them is measured. The proposed methodology takes this difference as an indicator of the severity of the disease; the smaller this difference, the more the white pixels increase, as does the severity of the disease. This difference is classified into five levels, with the fifth level indicating the highest degree of severity. At this level, there is little difference between the numbers of black pixels in the original image and the multi-colored image. The first level means that the virus does not control the lung; in this case, there is a noticeable difference between the numbers of black pixels in the images.

### 2.2. Data Analysis

Three independent radiologists (each at least with 4 years of experience reporting various chest X-ray cases) were heavily involved in the process of assessing the recruited patients’ medical records and the provided chest X-ray images. The Picture Archiving and Communication System facilities were used to report the data. A classification of five levels of severity was based on data obtained from chest X-ray images and the radiologists’ evaluation in comparison to the patient’s history.

**Figure 2:**
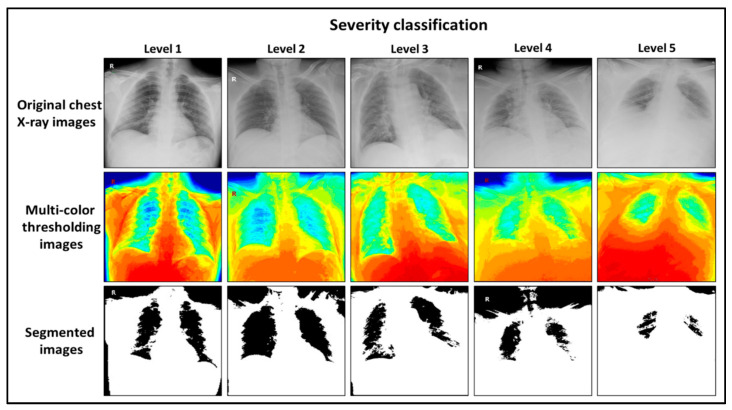
Chest X-ray images for five confirmed SARS-COV2 cases. These images show the levels of severity of inflammation in three different modes: original image mode (first row), multi-color thresholding mode (second row), and segmentation mode (third row).

Based on the study of the patients’ symptoms and findings from their chest X-rays, the first level indicates the lung with limited signs of inflammation, as seen in Figure 2. Patients in this level had symptoms such as a high temperature but no cough or signs of difficulty breathing. Patients in the second level had mild lung inflammation, a high temperature, and mild coughing but no signs of difficulty breathing. Patients in the third level had moderate inflammation in the lungs, moderate coughing, shortness of breath, and pneumonia. Patients in the fourth level experienced advanced symptoms, such as critical inflammation in the airways and lungs, a lack of oxygen in the blood, acute respiratory distress, and complications of the immune system and other organs. Patients in the fifth level were disabled by life-threatening lung infections, with serious tissue inflammation, requiring the patient to be placed on artificial ventilation.

Various quantitative measures were used to analyze the data: the numbers of cumulative ratios of white pixels to black pixels (mean, standard deviation, *t*-test, *p*-value, and Cohen’s Kappa). These statistical parameters were be used to measure the degree of convergence between the results of the proposed methodology and the opinions of experienced radiologists in classifying X-rays into the above-mentioned severity classification.

## 3. Results and Discussion

Figure 2 shows examples of the five levels of severity that the methodology suggests. They are based on chest X-ray images for confirmed COVID-19 patients. As shown in the figure, the degree of severity classification for each level is shown in three ways. In the first row, the X-rays are classified according to the volume of the appeared lung. In the original chest X-ray image, the volume of healthy lung tissue can be determined by calculating the ratio of dark pixels (which express healthy tissue) to bright pixels (which express tissue affected by the disease): the higher the percentage, the lower the risk ratio, and vice versa. Furthermore, since the volume of a healthy lung reflects the extent to which the patient is affected by the virus, volume directly reflects the degree of severity; decreased lung volume indicates greater severity. In the second row, the X-ray images have been processed and converted into a 19-color primary image. This image has the advantage of presenting the lung and its surroundings in 19 layers, which gives unconventional features to these images (i.e., when the image is divided into layers, the boundaries of each layer are clearly visible, which helps accurately define the areas and locations of healthy lung tissue). In the third row, the segmented X-ray images show the remaining parts of the healthy lung, or the parts of a patient’s lungs that are inflammation-free.

**Figure 3:**
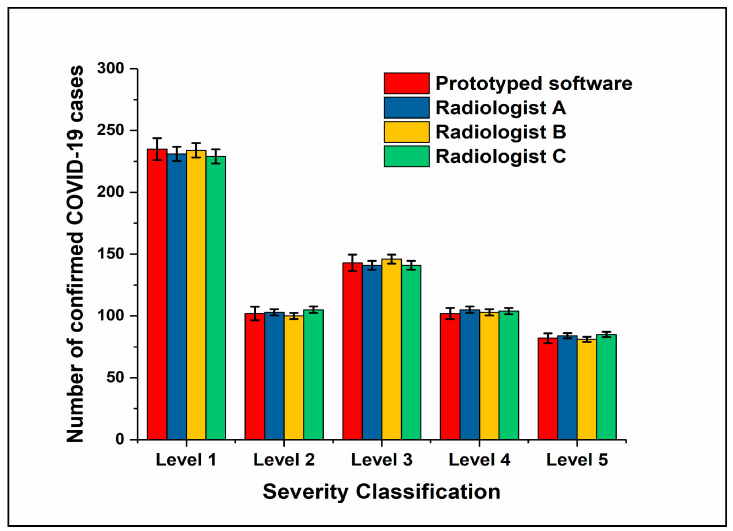
Evaluation data provided by three radiologists compared to the prototype color-thresholding auto-detection method.

Figure 3 is a benchmark comparison of the results of the proposed computational methodology and the classifications by the three radiologists of the collected datasets. The results of the benchmark comparison between the results of the proposed methodology and the radiologists’ evaluation converged in their diagnosis of the five levels, with a rate of 91.5%.

**Figure 4:**
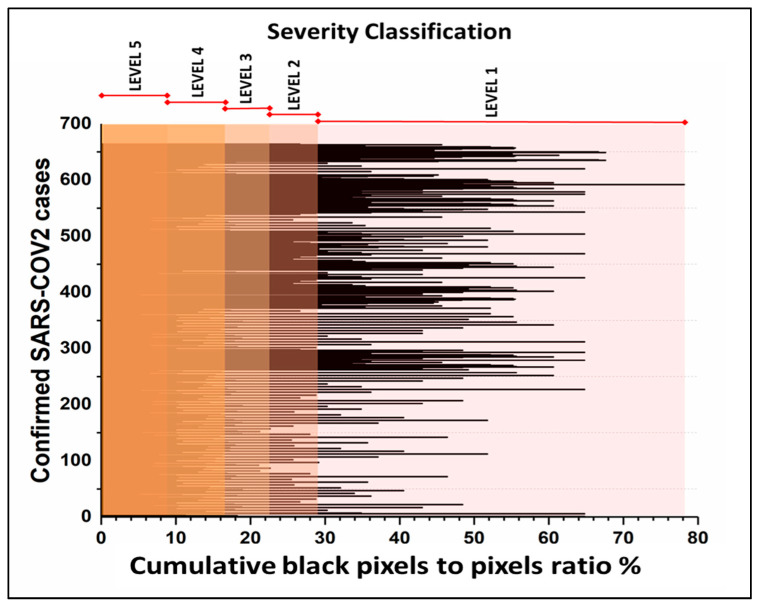
Classifications of severity by the prototype color-thresholding auto-detection method. The distribution of the data shows the ratio of cumulative black pixels to total pixels in the ROI.

Figure 4 is a frequency graph that shows the severity of each subject in this study, based on which the number of each patient in each severity level was determined. The results show that if the ratio of black pixels to white pixels is close to 45%, this indicates level one; a ratio close to 27% indicates level two; a ratio of 20% indicates level three; a ratio of 17% indicates level four; and a ratio of 4% indicates level five.

**Figure 5:**
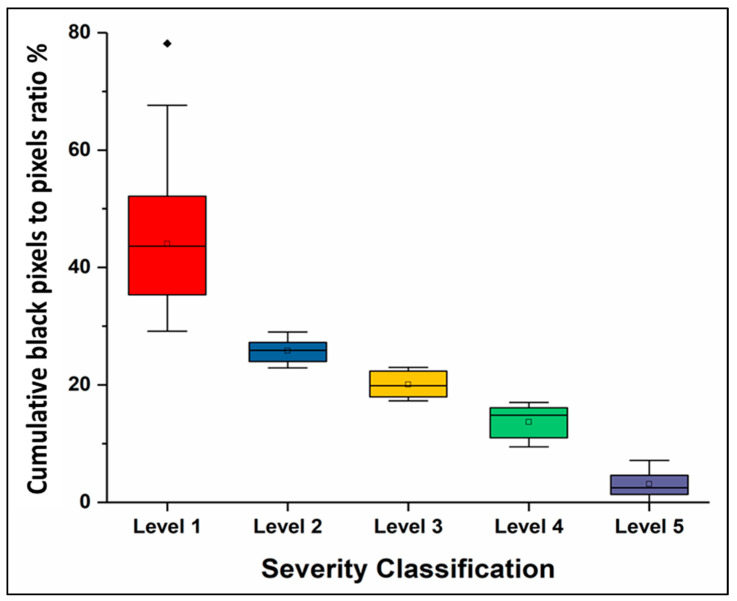
Box plot represents the severity levels of the disease based on the data retrieved from the X-ray images and the cumulative black pixels to total pixels in the ROI.

Figure 5 is a box plot that represents the severity levels of the disease based on the data retrieved from the X-ray images and the cumulative black pixels to total pixels in the region of interest (ROI), or the region in a set of samples within a data set that has been identified for a specific purpose. In the case of this work, the ROI is the shadow of the lungs in the chest X-ray image. Table 1 presents the quantitative data for the ratio of cumulative white pixels to black pixels against the gold standard (i.e., radiologists’ evaluation). It is concluded from the analysis that the central trend of cases is to the first level of severity. The standard deviation is low, meaning that the data is clustered around the mean. The *p*-value is 0.27, which indicates an insignificant statistical difference between the outputs of the suggested technique and the radiologist outputs. Cohen’s Kappa is between 0.81 and 1.00, implying that there is complete harmony between the performance of the proposed technique and the radiologists.

The outcome of this study has been compared with comparable published studies using novel methods for evaluating chest X-ray images; Table 2 provides the details of detection accuracy for these computational models. The proposed novel computational model could be used for the quantification of COVID-19 and the critical decision-making process to provide appropriate follow-up interventions with patients who require it. Although the proposed model may not eliminate conventional diagnostic techniques, it may be used to complement such techniques and reduce testing by providing services to patients in need of emergency care. The method may also be used in situations in which chest X-ray images require further evaluation by medical specialists.

The literature contains different novel methods to detect and evaluate chest X-ray images for COVID-19 patients with high accuracy levels (i.e., >90%); nevertheless, some of these methods have been built in small datasets (i.e., <100 chest X-ray images), which may need bigger sample size to validate its accuracy [37]. Furthermore, some of the recently published methods were limited in providing information regarding the disease severity [38], which could require more investigations and advanced computational methods to comprehensively evaluate patients’ condition in a more specific manner.

## 4. Conclusions

The aim of this research has been focused on finding an effective and accurate method for identifying the location of effected regions in the lungs of confirmed COVID-19 patients and classifying the disease severity using conventional chest X-ray images. The proposed methodology divides each image into multiple layers, analyses each layer, and then classifies the disease into five levels of severity. This analysis identifies locations of affected regions in the lungs and indicates the degree of severity of the disease by comparing affected regions with healthy tissue. The results of this methodology were compared with the opinions of experienced radiologists in evaluating X-ray images, and the methodology matched these opinions at a rate of 91%. Thus, this research provides an accurate method of identifying inflammation sites caused by COVID-19, which may enable care providers to quickly implement effective methods of treatment.

## Figures and Tables

**Table 1:** Quantitative statistical evaluation for the outcome of the proposed model against radiologists’ readings.

Statistical Parameters	Value	Conclusion
Standard deviations	1.1428	This value is tiny, indicating that the data are clustered in the center.
*t*-test	0.9319	Test values < 1 mean that the outputs of the proposed technique and the radiologists are not substantially different.
*p*-value	0.2738	A *p*-value > 0.05 means that the outputs of the proposed auto-detection model is not significantly different from those of the radiologists.
Cohen’s Kappa	0.9141	This value varies from 0.81 to 1.00. This ensures that the findings of the proposed technique and the radiologists are perfectly compatible.

**Table 2:** Accuracy comparison for the proposed model and other published models for a similar purpose.

Study	Year	Accuracy
Cohen et al. [36]	2020	80%
Amer et al. [37]	2020	94%
Afshar et al. [38]	2020	96.24%
Borkowski et al. [39]	2020	89%
Harmon et al. [40]	2020	90.8%
Snider et al. [41]	2020	90.56%
Proposed method	2021	91%

## Data Availability

Not applicable.
